# Grafted Pullulan Derivatives for Reducing the Content of Some Pesticides from Simulated Wastewater

**DOI:** 10.3390/polym14132663

**Published:** 2022-06-29

**Authors:** Luminita Ghimici, Marieta Constantin, Maria-Magdalena Nafureanu

**Affiliations:** “Petru Poni” Institute of Macromolecular Chemistry, Aleea Grigore Ghica Voda 41A, 700487 Iasi, Romania; marieta@icmpp.ro (M.C.); nafureanu.magda@icmpp.ro (M.-M.N.)

**Keywords:** pullulan-based flocculant, insecticides, synthetic wastewater, flocculation mechanism, UV-Vis spectroscopy

## Abstract

The goal of the current article was to obtain data regarding the application of a series of grafted pullulan derivatives, as flocculating agents, for removal of some pesticide formulations from model wastewater. The pullulan derivatives are cationic polyelectrolytes, with various content and length of grafted poly[(3-acrylamidopropyl)-trimethylammonium chloride] chains onto the pullulan (P-*g*-pAPTAC)]. The commercial pesticides are either fungicide (Bordeaux Mixture) (*BM*) or insecticides (Decis (*Dc*)—active ingredient Deltamethrin, Confidor Oil (*CO*)—active ingredient Imidacloprid, Confidor Energy (*CE*)—active ingredients Deltamethrin and Imidacloprid and Novadim Progress (*NP*)—active ingredient Dimethoate). The removal efficiency has been assessed by UV-Vis spectroscopy measurements as a function of some parameters, namely polymer dose, grafted chains content and length, pesticides concentration. The P-*g*-pAPTAC samples showed good removal efficacy at dose_op_, more than 94% for *BM*, between 84 and 90% for *DC*, *CO* and *CE* and around 93% for *NP*. The maximum percentage removal decreased with the pesticides (*DC*, *CO*, *CE*, *NP*) concentration declining; no effect of *BM* concentration in suspension on its removal efficiency process has been noted. Differences indicated by zeta potential and particle size distribution measurements regarding the pesticides removal mechanisms by pullulan derivatives (charge neutralization, bridging, etc.) are discussed.

## 1. Introduction

Graft copolymers are compounds obtained by one of the widely used chemical modification method of synthetic or natural polymers, namely graft copolymerization one, with the three synthesis strategies (the “grafting onto”, the “grafting from”, and the “grafting through”) [1]. The possibility of combining a large number of monomers and polymers has been materialized in obtaining compounds with tailored compositions (functional groups type, grafting density, side/graft chains lengths, etc.), and hence improved or new properties suitable for a wide range of applications in various industrial, biomedical, pharmaceutical, agricultural, environmental fields, etc. [2,3,4]. Over time, many researchers have focused on the synthesis and characterization of soluble grafted polysaccharides used in the wastewater treatment processes, the interest in developing these materials being prompted by the possibility to combine the advantages of polysaccharides (cheap, non-toxic, biodegradable and fairly shear stable) [5] and those of synthetic polymers (low dosage). Thus, many grafted copolymers of chitosan, cellulose, starch, konjac glucomannan, gum guar, gum tragacanth, alginate etc. have been synthetized and used for removal of clays, dyes, metal ions, etc. [3,5,6,7,8,9,10]. Few grafted copolymers have been used for adsorption of some pesticides from aqueous medium [11,12]. It is well known that this type of refractory contaminants used, especially, in the agriculture field to increase world food production have been a worldwide concern as a result of their undesired consequences (toxic effects) on the environment (soil, air, water) [13] and living organism health [14]. Therefore, the reduction of pesticide level in surface and wastewater resulting from pesticide production plants and agricultural activities by some physical, chemical and biological methods has been given lots of attention [15,16,17,18,19]. In recent years, polysaccharide derivatives-based flocculation method has been used with good results to reduce the content of some fungicides and insecticides commercial formulations from the synthetic wastewater (removal efficiency between 90 and 97%). Polysaccharide derivatives were based on chitosan [20], dextran [21] and pullulan [22]. Regarding pullulan and its derivatives, the literature survey of some valuable reviews [5,22,23] revealed that these compounds, including the grafted ones were less tested as flocculant [24,25], in spite of the high flexibility of the pullulan backbone which afford a suitable arrangement of the polymer chains on the particles surface. In addition, the ionic derivatives have the advantage of charged groups presence, able of electrostatically attracting charges from the surface of contaminant particles. The excellent properties mentioned above have been recently demonstrated by some pullulan derivatives containing either pendent tertiary amine groups or quaternary ammonium salt one (grafted chains onto the pullulan), which have been tested and proved to be very efficient in separation of kreutzonit particles and their mixture with kaolin, K-feldspar, hematite (95-99% in the optimum dose domain) [26]. Moreover, the flocs resulting from the separation of kreutzonit particles by the pullulan derivative sample with grafted cationic chain (poly[(3-acrylamidopropyl)-trimethylammonium chloride]) (P-*g*-pAPTAC) reduced successfully fungicide Bordeaux mixture (BM) from synthetic wastewater (removal efficacy more than 95%). This result led us to question whether the soluble P-*g*-pAPTAC samples containing various amount and length of grafted cationic chains could be also effective in removing BM, but also other commercial pesticide formulations from simulated dispersions. The answer was found in the present investigation that, mainly, considered the impact of the pullulan derivatives chemical structure (grafted chains content and length) and polymer dose (the flocculant concentration in its mixture with pesticide dispersions) on the removal of some commercial insecticide formulations Decis (*Dc*) (Delthamethrin—active ingredient), Confidor oil (*CO*) (Imidacloprid—active ingredient), Confidor Energy (*CE*) (Deltamethrin and Imidacloprid—active ingredients), Novadim Progress (*NP*) (Dimethoate-active ingredient) and fungicide *BM* (copper ion as copper sulfate). To the best of our knowledge, there have been no reported data regarding the impact of grafted pullulan derivatives, as purification agents in pesticide-containing wastewater.

The UV–Vis spectroscopy together with the zeta potential and particle aggregates size measurements were the tools used to determine the separation efficiency and the flocculation mechanism for each pesticide investigated.

## 2. Materials and Methods

### 2.1. Materials

**Figure 1:**
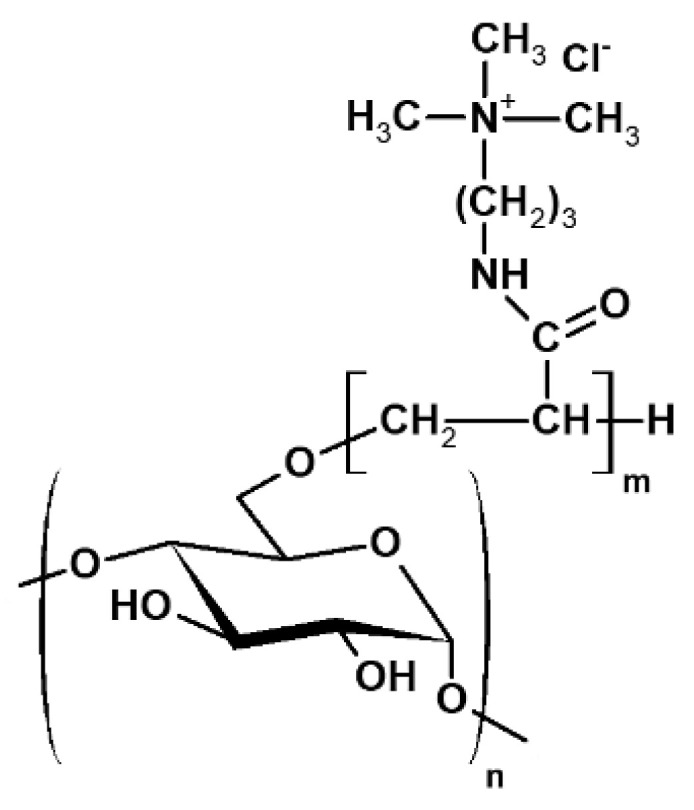
General chemical structure of polycations based on pullulan **P-*g*-pAPTAC**.

*Pullulan derivatives samples* (P-*g*-pAPTAC) with various amount and length of grafted cationic chains, were obtained by free–radical grafting of (3-acrylamidopropyl)-trimethylammonium chloride (APTAC) onto pullulan (Mw = 200 kg mol^−1^) (Hayashibara Lab. Ltd., Okoyama, Japan), in the presence of initiator potassium peroxydisulfate, as it was described by Constantin et al. [27] (Figure 1).The polymers abbreviations are given in the footnote of Table 1 which collects the synthesis parameters and some characteristics for the pullulan derivatives.

*Pesticides: Bordeaux mixture* MIF type (IQV, Barcelona, Spain) (*BM*)—commercially accessible in packs of 50 g. *Decis* (Bayer CropScience, Leverkusen, Germany) (*Dc*)—commercially accessible in vials with 2 mL solution. *Confidor Oilsc_0.04_* (*CO*) (Bayer, Leverkusen, Germany) and *Confidor Energy* (*CE*) (Bayer)—commercially accessibles in bottles with 100 ml concentrated suspension. *Novadim Progress* (*NP*) (Cheminova A/S, Lemvig, Denmark)—commercially available in vials with 20 mL solution. The chemical structure of active ingredients for each pesticides formulation as well as some other their characteristics and of model pesticides dispersions are shown in Table 2.

### 2.2. Methods

The stock solutions of the grafted pullulan derivatives were prepared in distilled water (concentration: 1 gL^−1^). They were stabilized at room temperature for one day before use. The pesticide dispersions, with characteristics indicated in Table 2, were also prepared in distilled water and stabilized by sonication for 15 min (ultrasonicator VCX 750 SONICS, Newtown, CT, USA) before starting tests. A Cole Parmer Nine-Position Stirring Hot Plate was used for assessing the polycations based on pullulan, as flocculants, in aqueous pesticide dispersions. The flocculation tests were carried out according to Ghimici and Nichifor [29]. Thus, the addition of pullulan derivatives to simulated dispersions of pesticides (50 mL placed into 100 mL beakers) took place under stirring at a speed of 500 rpm, which was kept constant for another 3 min. Afterwards the speed was decreased to about 200 rpm for 15 min. The flocs were then allowed to settle down. At the end of the optimum settling period fixed for each particle (the period of time after which the pollutants residual absorbance (%) remained almost constant), absorbance measurements (spectrophotometer SPECOL 1300 (Analytik Jena GmbH, Jena, Germany)) at λ values mentioned in Table 2 and zeta potential ones (Zetasizer Nano-ZS, ZEN-3500 model, Malvern Instruments, Malvern, England) were performed on supernatant samples (10 mL). The optimum settling time for each pesticide formulation was established in preliminary experiments, as follows: 60 min for *BM*, 1200 min for *CO*, *Dc*, *CE* and 120 min for *NP*. Also, to evaluate the "natural" separation of the dispersions, blank tests were carried out on pesticide dispersions without pullulan derivatives. Thus, the residual absorbance values were 90.16% for *BM*, 92.5% for *CO*, 90% for *CE*, 85% for *Dc* and 80% for *NP* after the same settling time as that established in the presence of polymers. The fungicide removal efficacy was expressed as percent of the initial absorbance recorded for the fungicide particles suspensions, at time zero (without polymer).

The size distribution measurements of the insecticide particles in initial dispersion and of polymer/pesticide aggregates at dose_op_, have been also carried out with Laser Particle Size Analyzer—Partica LA-960V2 (Horiba, Kyoto, Japan) (D(50), µm).

## 3. Results and Discussion

### 3.1. Effect of Polymer Dose and Grafted Chain Content and Length

#### 3.1.1. Fungicide Bordeaux Mixture

*BM*—a combination of copper sulfate, lime, and water is an effective bactericide and fungicide that provides a long-lasting protection to fruit trees, ornamental plants, vine fruits, etc. [30]. However, the excessive use of *BM* is risky, as it can be toxic to livestock, earthworms, fish, and even humans [31,32,33]. Hence, the reduction content of copper and even elimination from soil and water is very important. In a comprehensive review, Al-Saydeh et al. (2017) [34] have focused on various treatment methods (physical, chemical and biological) of wastewater contaminated with copper. Also, Oustriere et al. [35] treated *BM* effluents by rhizofiltration in constructed wetlands (pilot-scale). Recently, the flocculation method was used with very good results for the removal of *BM* particles from simulated wastewater in the presence of some polysaccharide derivatives, pullulan with pendent tertiary amine groups [36] and chitosan [20].

**Figure 2:**
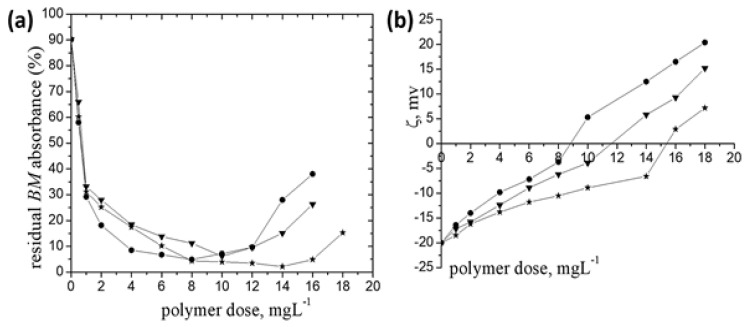
The residual *BM* absorbance (%) (**a**) and zeta potential (ζ) (**b**) dependence on the polycation dose: **P-*g-*pAPTAC1** (inverted triangle), **P-*g*-pAPTAC2** (star), **P-*g*-pAPTAC3** (circle); c_BM_ (%, *w*/*w*)—0.05, settling time 60 min.

In the following, the effects of flocculant dose and of the grafted pAPTAC content and length in the pullulan derivatives on the removal efficiency of *BM* are shown in Figure 2.

A maximum efficacy in removal of BM (around 94% and more) was noticed for the samples investigated at optimum polymer doses (dose_op_—the polymer dose corresponding to the maximum removal efficiency of particles). The explanation for this result can be found below. In order to be a good flocculant, a polymer must be adsorbed on the surface of the particles by means of some forces such as hydrogen bonding and electrostatic attractions and/or hydrophobic ones and ion binding [37]. The conformations of the adsorbed chains (loops, trains, and tails) resulted as a consequence of the polymer/particles interactions mentioned above lead to various flocculation mechanisms, such as: bridging (where tails and loops of a few polymers with high affinity to the particle surface make bridges between two or more particles), charge neutralization (where the particle surface charges are neutralized by the oppositely charged groups of the macromolecular chain so that the particles attract each other by van der Waals forces), or a charge patch mechanism (when aggregation occurs as a result of the electrostatic attraction between oppositely charged regions on partially covered particles) [37]. Quite often these separation mechanisms act in combination, depending on the properties of particles and polymers. The grafted pullulan derivatives studied herein, contain quaternary ammonium salt groups which can electrostatically attract the SO_4_^2−^ anions (the negative species of this fungicide (ζ = −20 mV)) inducing, thus, the *BM* particles aggregation and settling. On the other hand, the chemical structure (Figure 1) shows that these polymer samples contain amide groups which can bind Cu^2+^ ions. Consequently, the polyions/Cu^2+^ ions interactions can have some contribution in the *BM* separation process. However, in the case of all P-*g*-pAPTAC samples an increase of the residual absorbance at polymer dose higher than dose_op_ has been observed; this could happen as at overdose the surface *BM* particles is less charged, and hence a high number of charges on the P-*g*-pAPTAC chains remain uncompensated leading to restabilization of suspension as an effect of the electrostatic and/or steric chain repulsions.

The results have also indicated that there was a difference between polyelectrolyte amount required for the maximum *BM* particles removal (Figure 2a). For the same grafted chain length, the sample with the highest ionic groups content, P-*g*-pAPTAC3 (pAPTAC (wt %) = 34.51, see Table 1) accomplished the lowest residual *BM* absorbance (4.9%) at dose_op_ of 8 mg·L^−1^, as against P-*g*-pAPTAC1 (pAPTAC (wt %) = 22.53), where the minimum residual *BM* absorbance (6.18%) was noticed at dose_op_ of 10 mg·L^−1^; the higher pAPTAC content determined the increase polyions/SO_4_^2−^ anions interactions and hence a lower dose_op_ for P-*g*-pAPTAC3. As regard P-*g*-pAPTAC2, a percent *BM* removal more than 95% has been observed in a large dose_op_ interval (between 8 mg·L^−1^ and 16 mg·L^−1^). This sample contains the longest cationic pAPTAC chains grafted on pullulan backbone, and hence a larger hydrodynamic coil volume than the other two samples (see the [*η*] values in Table 1). This implies a more facile accessibility, and consequently a higher number of attached fungicide particles to the positive sites of the polymer chain. The binding of more particles by a polymer chain is characteristic, as it is already mentioned, for the bridging mechanism [37] which has to be taken under consideration for this system. On the other hand, the smaller number of cationic positions uninvolved in interactions with the *BM* particles, may cause poorer repulsive interactions between the polyion segments, and hence the lagging redispersion.

**Figure 3:**
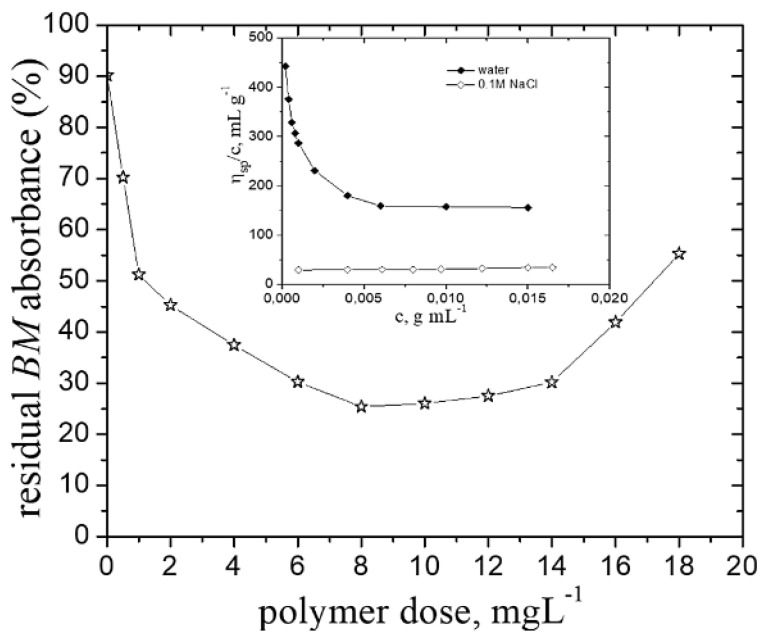
The residual *BM* absorbance (%) dependence on the polymer dose (salt solution of P-*g*-pAPTAC2 in 0.1 M NaCl); *c*_BM_ (%, *w*/*w*)—0.05, settling time 60 min. The inset: the reduced viscosity dependence on polymer concentration, *c*.

The zeta potential measurements have provided information regarding the separation mechanism (Figure 2b). Kleimann et al. [38] have found that ζ value near zero at dose_op_ corresponds to the charge neutralization mechanism. Accordingly, the value of ζ = −3.69 mV (at dose_op_) pleads for the mechanism mentioned above, as the predominant one involved in the separation of *BM* particles by P-*g*-pAPTAC3. In case of P-*g*-pAPTAC2, the ζ measurements recorded values between −10.5 mV and 2.9 mV in the optimum dose interval; this confirms the UV-Vis measurements data, namely that alongside charge neutralization mechanism and the polyions/Cu^2+^ ions interactions, the bridging mechanism could have a noteworthy implication in the *BM* removal process. This was also checked by evaluation of the BM particle separation by a solution of P-*g*-pAPTAC2 prepared in 0.1 M NaCl (Figure 3). It is well known that the addition of an excess of salt in a polyelectrolyte solution leads to the screening of charged segments, its viscometric behavior in solution becoming similar to that of neutral polymers [39]. This happened for P-*g*-pAPTAC2 in solution of 0.1 M NaCl, when the reduced viscosity values (*η_sp_/c_p_*) decreased linearly with dilution (Huggins plot) (the inset of Figure 3).

A significant decline of the residual fungicide particle (%) in the presence of salt solution of P-*g*-pAPTAC2 was noticed, a maximum removal efficiency of around 70% being achieved in the dose_op_ interval between 6 mg·L^−1^ and 14 mg·L^−1^. This finding sustains the assumption above related to the predominant involvement of the bridging mechanism in the removal of *BM* particles by the pullulan derivative with the longest grafted chains. The implication of this type of flocculation mechanism in case of neutral grafted polymers was previously reported [5,40].

One has also to stress that NaCl had no influence on the separation of this fungicide; the suspension of *BM* particles prepared in 0.1 M NaCl solution was stable, a residual *BM* absorbance of 87% after 60 min of settling time being observed.

In closing this discussion, one may remark that the flocculation performance of the grafted pullulan derivatives with strong basic quaternary ammonium salt groups is quite close (removal efficiency of 94% and more in the dose_op_ interval between 8 mg·L^−1^ and 16 mg·L^−1^) to that recorded in case of the pullulan derivatives containing pendent tertiary amine groups, (separation efficacy of around 98% in the dose_op_ interval between 3 mg·L^−1^ and 20 mg·L^−1^) [36]. The difference lies in the settling time after which these results were obtained, namely 60 min for the former type of pullulan derivatives and 1200 min for the latter one. We assume that both the quaternary ammonium salt groups and grafted chains presence in the chemical structure of P-*g*-pAPTAC samples could determine an intensification of the polycation/*BM* particles interactions, and hence a more rapid separation of fungicide. Thus, the grafted pullulan samples could be used in the separation processes where a shorter settling time is preferred.

#### 3.1.2. Insecticides Decis, Confidor Oil, Confidor Energy

The *CO* and *Dc* formulations are systemic insecticides employed for the control of sucking insects (termites, thrips, aphids, etc) in crops of rice, cereal, vegetables, fruits, cotton, etc [41]. The active ingredients of these pesticides are Imidacloprid (1-(6-chloro-3-pyridyemethyl)–*N*-nitroimidazolidine-2-yliedeneamine) (neonicotinoids chemical family [42]) for *CO* and Deltamethrin ([(S)-Cyano-(3-phenoxyphenyl)-methyl] (1R,3R)-3-(2,2-dibromoethenyl)-2,2-dimethyl-cyclopropane-1-carboxylate) (pyrethroid chemical family) for *Dc*. These insecticides can be applied as single substance but also as mixture, for example in CE formulation which contains different amounts of both Imidacloprid and Deltametrin (see Table 2).

**Figure 4:**
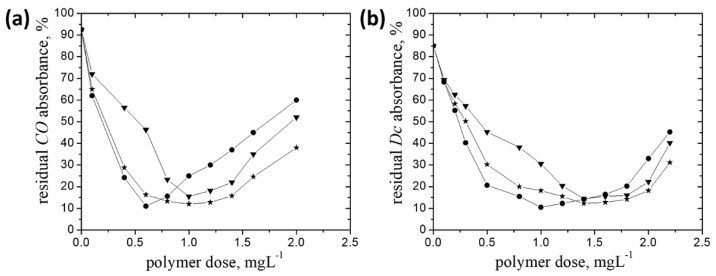
The residual pesticides absorbance (%) dependence on the polycation dose: **P-*g-*pAPTAC1** (inverted triangle), **P-*g-*pAPTAC2** (star), **P-*g-*pAPTAC3** (circle) for *CO* (**a**) and *Dc* (**b**); settling time 1200 min; *c_CO_* (%, *w*/*w*)—0.1; *c_Dc_* (%, *v*/*v*)—0.04, settling time 1200 min.

**Figure 5:**
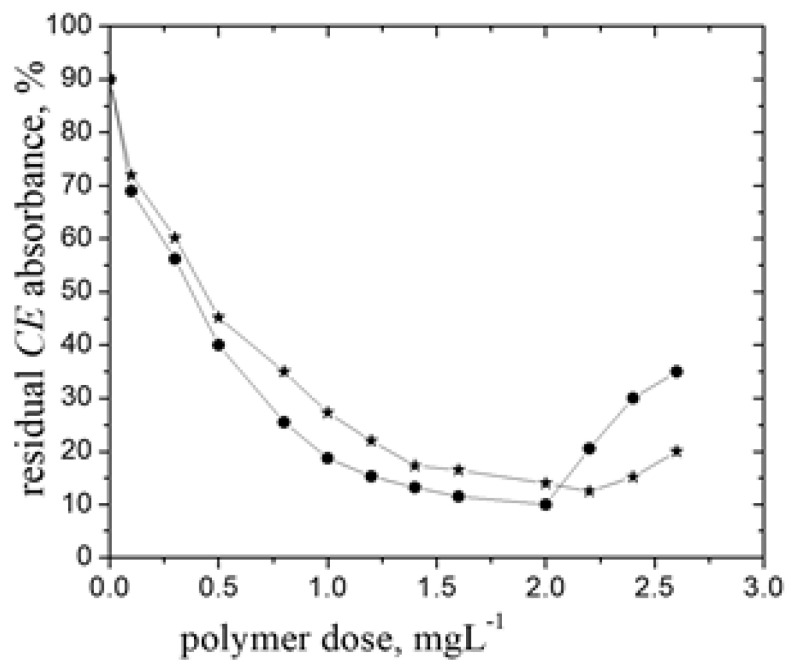
The residual CE absorbance (%) dependence on the polycation dose for **P-*g-*pAPTAC2** (star), **P-*g-*pAPTAC3** (circle); *c_CE_* (%, *w*/*w*)—0.03, settling time 1200 min.

The data showing the effect of grafted pullulan derivatives dose on the percent removal of the insecticides mentioned above are represented in Figure 4a,b and Figure 5.

In case of the pesticide formulations containing a single active ingredient, the following aspects can be highlighted: (i) a rise of the insecticides removal efficiency with increasing grafted pullulan derivatives dose, achieving the maximum at dose_op_ which depended on the ionic groups content; the higher the pAPTAC content, the lower dose_op_, as follows: dose_op_ (mg·L^−1^): 0.6 (P-*g*-pAPTAC3) against 1 (P-*g*-pAPTAC1) for *CO* and 1 (P-*g*-pAPTAC3) against 1.4 (P-*g*-pAPTAC1) for *Dc*; (ii) for both insecticides, no effect of the grafted chain length in P-*g*-pAPTAC2 on the doseop was observed, the values being located in the same interval as the other two polymers, namely 1 mg·L^−1^ for *CO* and 1.4 mg·L^−1^ for *Dc*. The findings above lead to the assumption that the electrostatic attractive interactions between the cationic sites on the polymer chains and the negative charged insecticide particles (ζ (*CO*) = −29.3 mV; ζ (*Dc*) = −28.2 mV), which are an indication for the charge neutralization or charge patch mechanisms, play the dominant role in the removal process. This assumption was checked by the zeta potential measurements, as in the case of *BM*. Looking at the experimental data in Table 3, one observes that for each pullulan derivative/insecticide system, the ζ values corresponding to dose_op_ are located around to zero.

Since P-*g*-pAPTAC1 was slightly less efficient in removal of both insecticides, than P-*g*-pAPTAC2 and P-*g*-pAPTAC3 (see Tabel 3), the tests for *CE* removal have been accomplished using the last two pullulan derivatives (Figure 5).

As in the case of insecticides containing a single active ingredient, both polymers proved to be efficacy in reduction of *CE* content in emulsion, the maximum removal efficiency of 90% for P-*g*-pAPTAC3 and 87.5% for P-*g*-pAPTAC2 being noticed at dose_op_ values of 2 mg·L^−1^ (P-*g*-pAPTAC3) and 2.2 mg·L^−1^ (P-*g*-pAPTAC2).

#### 3.1.3. Insecticide Novadim Progress

**Figure 6:**
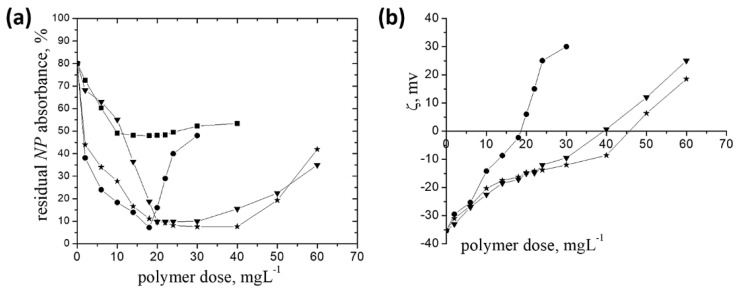
The residual NP absorbance (%) (**a**) and zeta potyential (ζ) (**b**) dependence on the polycation dose: **P-*g*-pAPTAC1** (inverted triangle), **P-*g*-pAPTAC2** (star), P-*g*-pAPTAC3 (circle); 0.1 M **NaCl P-*g*-pAPTAC3** (square); *c_NP_* (%, *w*/*w*)—0.7; settling time 120 min.

Novadim Progress is an organophosphorous insecticide - acaricide formulation with systemic action that acts on contact and ingestion [43], used in agricultural area to protect a wide range of crops (tomatoes, cabbage, cereals, fruits), tree and ornamentals from insect attacks [44]. Its active ingredient is Dimethoate ([O,O-Dimethyl S-(*N*-methylcarbamoylmethyl) phosphorodithioate]) which can undergo hydrolysis at the amide group [45], the insecticide particles gaining negative charges (*ζ_water_* = −35.3 mV). Thus, they could be able to interact electrostatically with the positive charges on the P-*g*-pAPTAC chains, the consequence being their aggregation and separation from the model emulsion, as illustrated in Figure 6a.

In addition, the hydrogen bonds formed between the amide groups of Dimethoate and of the pullulan derivatives could participate to the *NP* removal process.

A pronounced decrease of the *NP* content in the synthetic emulsion with the pullulan derivatives dose increase, up to 18 mg·L^−1^ (P-*g*-pAPTAC3), 22 mg·L^−1^ (P-*g*-pAPTAC1) and 30 mg·L^−1^ (P-*g*-pAPTAC2), when a high removal efficiency (between 90–93%) has been noted. On the other hand, the low residual *NP* absorbance (%) values, below 10 were observed on a larger flocculation interval for P-*g*-pAPTAC2 (20 mg L^−1^–40 mg L^−1^) against one doseop for P-g-pAPTAC3. The fastest separation of *NP* and, also, its rapid redispersion can be attributed to the enhanced content of cationic groups on the P-*g*-pAPTAC3 chain, as in case of the other pesticides already presented here. Both the high residual *NP* absorbance (48%) found when 0.1 M NaCl solution of P-*g*-pAPTAC3 was used as flocculant (the polymer becomes neutral as P-*g*-pAPTAC2 does—data not shown) and zeta potential measurements of *NP* emulsion as a function of polymer dose indicated that the separation process took place mainly by charge neutralization process (ζ value at dose_op_ = −2.3 mV) (Figure 6b).

The monotonous increase of ζ with polymer dose and the negative values in the optimum dose intervals obtained in the presence of P-*g*-pAPTAC1 (between −15 mV and −9.5 mV) and P-*g*-pAPTAC2 (between −14.7mV and −8.6mV) suggested us that the bridging mechanism and the hydrogen bonds established between the amide groups of both Dimethoate and the pullulan derivatives could become dominant in the separation process of *NP*.

From the data presented above, one may emphasize that the grafted pullulan derivatives are as good flocculants as other polysaccharides (chitosan [20] and dextran derivatives [21]) for *NP* particles (removal efficacy more than 90%). However, they are more suitable, especially P-*g*-pAPTAC2, in the flocculation processes where large dose_op_ intervals are required.

### 3.2. Effect of Pesticide Concentration

**Figure 7:**
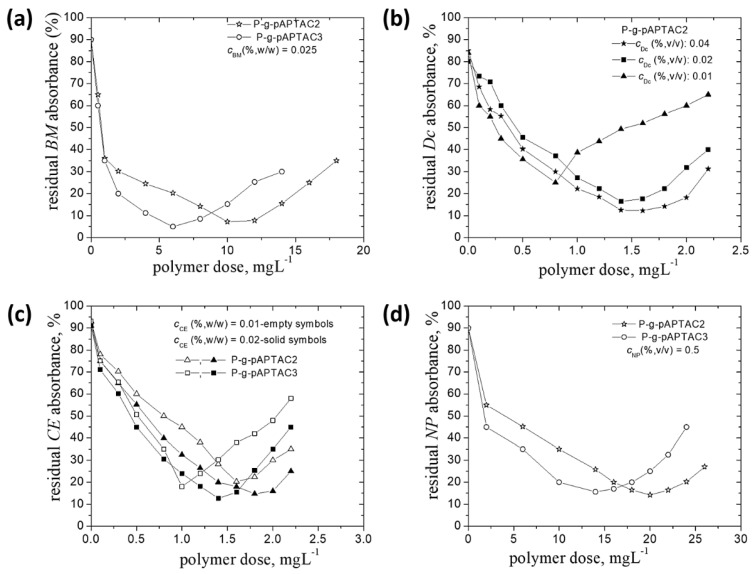
The residual pesticides absorbance (%) dependence on the polymer dose in dispersions with different concentrations of pesticides: *BM* (**a**), *Dc* (**b**), *CE* (**c**), *NP* (**d**).

Another important parameter which can have an impact on the removal efficiency of grafted pullulan derivatives is the amount of pesticides from wastewater. Hence, it is useful to perform experiments with dispersions containing different concentrations of pesticides (see Table 2). The results are plotted in Figure 7. P-*g*-pAPTAC2 and/or P-*g*-pAPTAC3 have been chosen in these experiments as they provided the best results in flocculation process, in terms of dose_op_ or percent of pesticides removal.

Both polymers showed the same behavior for all pesticides, at the new concentrations investigated, as that noticed for the already discussed concentrations, namely the lowest dose_op_ values were found for the P-*g*-pAPTAC3 sample and the largest dose_op_ intervals for P-*g*-pAPTAC2. However, there are some differences indicating the impact of the initial emulsions concentration on the removal pesticides efficiency. Thus, for the same pesticide, the dose_op_ values decreased with the decline of pesticide concentration (Table 4).

This likely occurred since a lower insecticide particles content in dispersion required less ionic polymer chains amount for the neutralization, hence the abatement of dose_op_. Another aspect which has to be underlined is the slightly decrease of the pesticide removal efficiency with reduction its concentration in dispersion from about 88% (c%, *v*/*v* = 0.04) to 75% (c%, *v*/*v* = 0.01) in case of *Dc* (see Figure 4b and Figure 7b), from 90% (c%, *w*/*w* = 0.03) to 82% (c%, *w*/*w* = 0.01) in case of *CE* (see Figure 5 and Figure 7c) and from about 93% (c%, *v*/*v* = 0.7) to 86% (c%, *v*/*v* = 0.5) for *NP* (see Figure 6 and Figure 7d). One may assume that a large distance between contaminant particles, at lower concentration, leads to a lower collision frequency and, thus, decreases the probability of their aggregation. A decrease of dose_op_ (mg L^−1^) with the decrease of *Dc* and *NP* concentration (%, *v*/*v*) has been noticed in the presence of a dextran derivative sample (D40-Et94), too: (dose_op_ = 1.4 (c_Dc_ = 0.04) to dose_op_ = 1.2 (c_Dc_ = 0.02) and dose_op_ = 6 (c_NP_ = 0.7) to dose_op_ = 4 (c_NP_ = 0.35) [21]. As regard *BM*, the decrease of its concentration in suspension had an insignificant influence on the flocculation efficiency, a removal percent of around 95% being recorded at both concentrations investigated (Figure 3 and Figure 7a).

### 3.3. Particle Size Measurements

**Figure 8:**
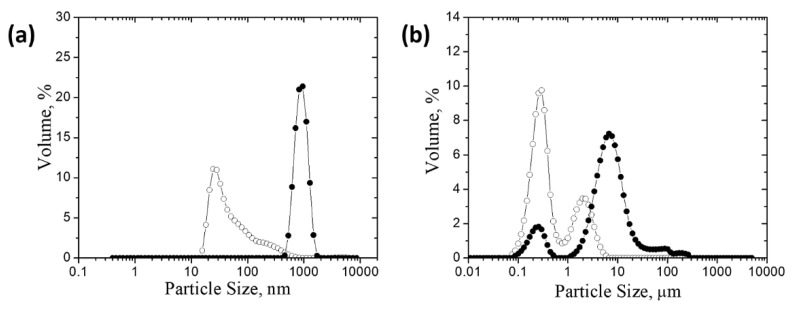
Particle size distribution for *NP* particles (**a**) and *BM* particles (**b**); initial pesticide particles (empty symbols); aggregates obtained in the presence of **P-*g*-pAPTAC3**, dose_op_ = 18 mg·L^−1^, (c%, *v*/*v* = 0.7) and in the presence of **P-*g*-pAPTAC2**, dose_op_ = 10 mg·L^−1^, (c%, *w*/*w* = 0.05) (solid symbols).

Both the Uv-Vis spectroscopy and zeta potential measurements have emphasized that the mechanisms of pesticide removal processes depend on the content and length of grafted cationic chains (pAPTAC). Thus, the pullulan sample with the highest charged groups content (P-*g*-pAPTAC3) accomplishes pesticides removal, mainly, through the neutralization mechanism while that with the longest grafted chains (P-*g*-pAPTAC2) through the bridging one. This finding has been enforced by the particle size measurements (D(50), µm) performed on the initial pesticide particles (before treatment with polymers) and aggregates obtained at the optimum polycation doses. As the curves describing the aggregate size distribution (volume fraction versus particle diameter), have not shown significant difference in shape, those revealing the results obtained in the removal of *NP* particles by P-*g*-pAPTAC3 and *BM* ones by P-*g*-pAPTAC2 are presented (Figure 8).

The untreated *NP* particles have a unimodal distribution (Figure 8a), with D(50) of 0.132 µm. This type of distribution was also maintained in case of the *NP* aggregates obtained in the presence of P-*g*-pAPTAC3; their narrow size distribution along with the small size (1.169 µm) strengthen that the charge neutralization prevails in the *NP* particles removal process. In case of fungicide dispersion, a bimodal size distribution of the *BM* particles has been recorded both in the absence and presence of P-*g*-pAPTAC2; the D(50) values were 0.308 µm and 5.697 µm for *BM* particles in the initial suspension and *BM*/P-*g*-pAPTAC2 aggregates, respectively. The high-volume percentage of the peak corresponding to particles of larger size confirms the assumption that the bridging mechanism could have the most important role in the *BM* particles separation process.

## 4. Conclusions

The commercial formulations of fungicide Bordeaux mixture *(BM*) and insecticides Decis (*Dc*), Confidor Oil (*CO*), Confidor Energy (*CE*) and Novadim Progress (*NP*) have been separated from the synthetic wastewater by aqueous solutions of grafted pullulan derivatives (P-*g*-pAPTAC) and the results can be resumed as follows:

The polymer doses required for maximum removal efficiency of the pesticides investigated shifted to lower values with augmentation of ionic groups content and abatement of pesticide concentration;

The longer the grafted chains, the larger the optimum dose interval, irrespective of the pesticide type;

Zeta potential data showed that (*i*) the neutralization mechanism prevails in case of *Dc* and *CO* particles removal by all of the pullulan derivatives as well as in case of *BM* and *NP* separation by the highest charged sample (P-*g*-pAPTAC3); (*ii*) the bridging mechanism has a noteworthy contribution in the *BM* and *NP* particles removal by the sample containing the longest grafted chain (P-*g*-pAPTAC2); (*iii*) the interactions of amide groups of the pullulan derivatives with (*1*) Cu^2+^ ions (of *BM*) and (*2*) the hydrogen bonds formed with those of Dimethoate (*NP*) could come into play in the separation process of these pesticides.

The good performance of the grafted pullulan derivatives in reducing the content of pesticides in wastewater is a reason for us to consider other parameters in future investigations (medium pH, mixture of pesticides as well as pesticides combined with other pollutants (salts, clays, etc.)).

## Figures and Tables

**Table 1:** Synthesis parameters of pullulan derivatives P-*g*-pAPTAC [25].

Polymer	p (*g*)	APTAC (·10^−2^ mol)	KPS (·10^−2^ mol)	Product		Mw ^2^ × 10^−3^ (g·mol^−1^)	[η] ^3^ _Rao_ (mL·g^−1^)
				pAPTAC (wt %)	Graft Ratio ^1^ (%)		
P-*g*-pAPTAC1	1.0	0.487	0.0369	22.53	29.09	13.81	67
P-*g*-pAPTAC2	1.0	0.967	0.0369	29.05	40.94	21.13	500
P-*g*-pAPTAC3	1.0	0.487	0.0924	34.51	52.69	33.28	77

**P** = pullulan, **APTAC** = (3-acrylamidopropyl)-trimethylammonium chloride, **KPS** = potassium peroxydisulfate, **pAPTAC** = grafted cationic chains, poly[(3-acrylamidopropyl)-trimethylammonium chloride] (pAPTAC). ^1^ Graft ratio is calculated with the equation: (weight of grafted polymer–weight of substrate)/weight of substrate; ^2^ Average molecular weight in 0.5 M NaCl at 25 °C; ^3^ [η]_Rao_ = the intrinsic viscosity determined by the Rao method (1993) [28] (see [25]).

**Table 2:** Pesticides and dispersions characteristics.

Pesticide	Chemical Structure	Chemical Composition (wt %)	Dispersion Concentration (c%, *w*/*w*)	Zeta Potential (ζ), mV	λ (nm)	pH
*BM*	-	(20% copper as copper sulfate)	0.05 0.025	−20	652	7
*CO*	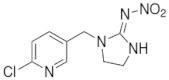	Imidacloprid: 4 g·L^−1^	0.1	−29.3	269	5
*DC* ^1^	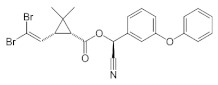	Deltamethrin: 50 g·L^−1^; solvent naphtha (petroleum), heavy arom.	0.04 0.02 0.01	−28.2	267	5
*CE*	-	Imidacloprid: 75 g·L^−1^ Deltametrin: 10 g·L^−1^	0.03 0.02 0.01	−29.9	270	5
*NP* ^1^	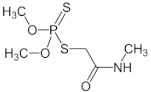	Dimethoate: 400 g·L^−1^; solvent cyclohexanone, xylened)	0.7 0.5	−35.3	601	4.5

^1^ c (%, *v*/*v*).

**Table 3:** Zeta potential (ζ) values corresponding to the polymer optimum dose (dose_op_).

Polymer Sample	*CO*			*Dc*		
	dose_op,_ mg L^−1^	Zeta Potential (ζ), mV	Removal Efficiency (%)	dose_op_, mg L^−1^	Zeta Potential (ζ), mV	Removal Efficiency (%)
P-*g*-pAPTAC1	1.0	−4.8	84.5	1.4	−5.2	85.5
P-*g-*pAPTAC2	1.0	+4.5	88	1.4	+4.5	87.7
P-*g*-pAPTAC3	0.6	−0.88	89	1	+0.2	89.5

Based on this finding, one may assert that the charge neutralization mechanism prevails in the removal of *Dc* and *CO* particles.

**Table 4:** Optimum dose corresponding to the pesticide dispersion concentration.

Polymer Sample	*BM*		*Dc*		*CE*		*NP*	
	Dispersion Concentration (c%, *w*/*w*)	dose_op,_ mg·L^−1^	Dispersion Concentration (c%, *v*/*v*)	dose_op,_ mg·L^−1^	Dispersion Concentration (c%, *w*/*w*)	dose_op,_ mg·L^−1^	Dispersion Concentration (c%, *v/v)*	dose_op,_ mg·L^−1^
P-*g*-pAPTAC2	0.05	14	0.04	1.6	0.03	2.2	0.7	30
	0.025	10	0.02	1.4	0.02	2	0.5	20
			0.01	0.8	0.01	1.6		
P-*g*-pAPTAC3	0.05	8	-	-	0.03	2	0.7	18
	0.025	6	-	-	0.02	1.4	0.5	14
					0.01	1		

## Data Availability

The data presented in this study are available on request from the corresponding author.
